# Impact of neurofibromatosis type 1 on quality of life using the Skindex-29 questionnaire quality of life in NF1

**DOI:** 10.1186/s13023-024-03078-0

**Published:** 2024-02-24

**Authors:** Ana M. Cieza Rivera, Carlos Lobato Fuertes, Tania Fernández-Villa, Vicente Martín Sánchez, Isis Atallah

**Affiliations:** 1 Faculty of Health Sciences, Department of Biomedical Sciences, Area of Preventive Medicine and Public Health, Universidad de León, León, Spain; 2 Ophtalmologic Department, Complejo Asistencial Universitario de León, León, Spain; 3 Research Group in interactions gene- environmental and health (GIIGAS), Institute of Biomedicine, University of León, León, Spain; 4 Epidemiology and Public Health Networking Biomedical Research Centre (CIBERESP), Madrid, Spain; 5 Division of Genetic Medicine, Lausanne University Hospital and University of Lausanne, Lausanne, Switzerland

**Keywords:** Quality of life, Skindex-29, Neurofibromatosis 1, Emotion, Symptoms, Functioning

## Abstract

**Background:**

Neurofibromatosis type 1 (NF1) is one of the most common RASopathies predisposing affected patients to melanic lesions and benign tumors. NF1 is associated with considerable esthetic and functional burden negatively affecting the patient’s quality of life (QoL). This study aims to assess the clinical features of NF1 patients and evaluate their impact on QoL. We identified NF1 patients from a public health database of a region in Spain. All patients underwent clinical and ophthalmological evaluation for NF1 features. We measured QoL using the Spanish version of the Skindex-29.

**Results:**

Forty patients fulfilled the NF1 National Institute of Health criteria when we recruited patients. The median age was 42.00 years (IQR 26.5 -53.75). The median total Skindex-29 score was 12.3 (IQR 5.9–22.4); (emotion: 15.0, IQR 5.0-37.5; symptoms 8.9, IQR 0.0-17.9 and functioning 8.3; IQR 0.5–18.3). Women and NF1 patients with lower educational levels were associated with poorer QoL scores. We identified itching and sleep troubles to influence NF1 patients’ QoL negatively.

**Conclusion:**

NF1 considerably influences the psychological well-being of NF1 patients. We observed that female and low-educated patients scored higher on the emotional dimension of the Skindex-29 and could, therefore, be more at risk of depression. We also pointed out some “minor symptoms” that negatively impact NF1 patients’ QoL such, as itching and sleep troubles which doctors could treat if sought by doctors.

**Supplementary Information:**

The online version contains supplementary material available at 10.1186/s13023-024-03078-0.

## Background

Neurofibromatosis type 1 (NF1; OMIM# 162,200) is one of the most common autosomal dominant disorders with a prevalence of one in 2,500 to 3,000 individuals. It is characterized by café au lait spots, axillary freckling, Lisch nodules, dermal or plexiform neurofibromas, skeletal dysplasia, and optic gliomas [1]. As *NF1* is a tumor suppressor gene, 99% of NF1 patients develop benign tumors such as cutaneous neurofibromas, starting from puberty and increasing in size and number with age, pregnancy, or stress [2–4] or plexiform neurofibromas which are congenital tumors that can bulk at any time during life [1]. NF1 patients are also at risk of developing malignancies such as neurofibrosarcomas, pheochromocytomas, or breast cancer. With a complete penetrance and a high variability, the progression of the disease is unpredictable [5, 6]. In addition to disease burden, NF1 patients may suffer from stigmatization due to the unesthetic aspect and the visibility of the lesions, which might influence their physical, emotional, and social well-being. Studying the effects of NF1 on various aspects of quality of life (QoL) is important to implement beneficial strategies to improve the QoL of NF1 patients. Researches have used several questionnaires to study the QoL of NF1 patients primarily by post, mail or online surveys (SF-36, Skindex-29, NF1-AdQoL, WHOQOL-100, WHOQOL-Bref, cNF-Skindex, EQ-5D-5 L, INF1-QOL, PedsQL, DLQI, BoN, CHQ-PF50, ITQOL… ) [7–22]. In those studies, the QoL of individuals with NF1 was systematically below the QoL of the general population.

The purpose of this study was to assess the QoL of a phenotypically and clinically well-described cohort of adult individuals with NF1 to evaluate the impact of the different symptoms in their QoL. We used the Skindex-29, a skin disease-specific QoL questionnaire [23] used worldwide.

## Methods

### Study population

**Fig. 1:**
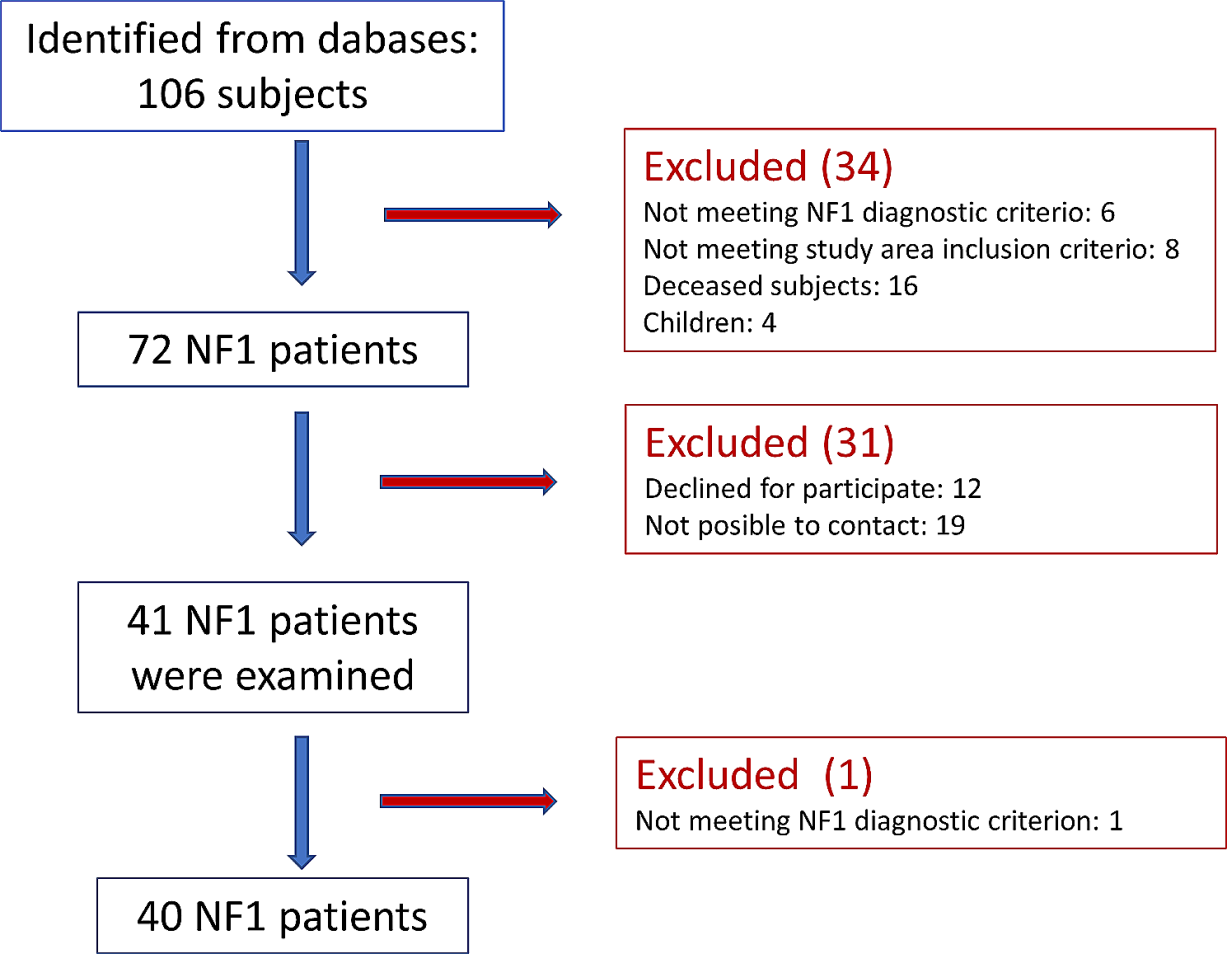
Flow diagram for patient recruitment

We identified NF1 patients by using the database of the Public Health Primary Care system and the database from the Leon main Hospital (Complejo Asistencial Universitario de Leon) by looking for the following items:

“*Neurofibromatosis*”, “*Neurofibromatosis type 1*”, “*dermal neurofibroma*”, " *plexiform neurofibroma*” *and* “*neurofibrosarcoma*”. We identified 106 patients fulfilling one of the mentioned criteria. We excluded patients with a diagnosis of neurofibromatosis type 2, carrying solitary tumors, or living outside the Leon public health. 16 patients were already deceased. We contacted patients by post and telephone. We could not get in contact with 18 patients and 14 patients refused to participate (Fig. 1).

We identified 45 patients with a diagnosis of NF1; we excluded four of them as children. Forty-one patients were enrolled in the study. NF1-expert clinicians and ophthalmologists examined all patients. A patient who did not fulfill the NF1 diagnosis criteria was excluded. Genetic analysis was available for 18 patients.

The Institutional Review Board and the Ethics Committee of Leon University Hospital approved the study protocol (approval number 1060).

### Severity and visibility evaluation

We assessed disease severity using the Riccardi scale [24], which has 4 degrees of severity, with Grade 1 being the mildest form and Grade 4 the more severe. In Grade 1, patients have some of the diagnostic features of NF1 without any compromising health and well-being. In Grade 2, patients have some features that make disease evident without impacting their health and well-being. In grade 3, patients have features that can impact their well-being without significantly compromising health, and in grade 4, patients have « seriously compromised health and well-being in a permanent, unmanageable way.».

We evaluated the disease visibility in full-dressed patients by using the Ablond scale [25] which has 3 degrees of severity. In grade 1, the disease is not visible with clothes. In grade 2, the patient presents some visible neurofibromas on the undressed body areas such as the face, neck, and hands or mild scoliosis. In grade 3, the disease is evident as the patient presents numerous visible tumors, disfiguring tumors, and severe complications such as severe scoliosis or cecity due to optic glioma.

### Quality of life measurement tool

The Spanish version of Skindex-29 [26], which the patient fulfilled during clinical evaluation, was used to measure the QoL of NF1 patients. The Skindex-29 has 29 items distributed in 3 domains which represent three specific aspects of skin disease: physical symptoms (items 1, 7, 10, 16, 18, 23 and 26), functioning (items 2, 4, 5, 8, 11, 14, 17, 19, 21, 24, 28 and 29) and emotions (items 3, 6, 9, 12, 13, 15, 20, 22, 25 and 27). Each item is rated on a 5-point Likert scale (never, rarely, sometimes, often, all the time). Scale scores were calculated by averaging the responses to items of a given domain. We standardized the scores to percentages. A higher score indicated a more significant effect of the disease. Individual results of Skindex-29 are available in Supplementary File 1.

### Data analysis

**Fig. 2:**
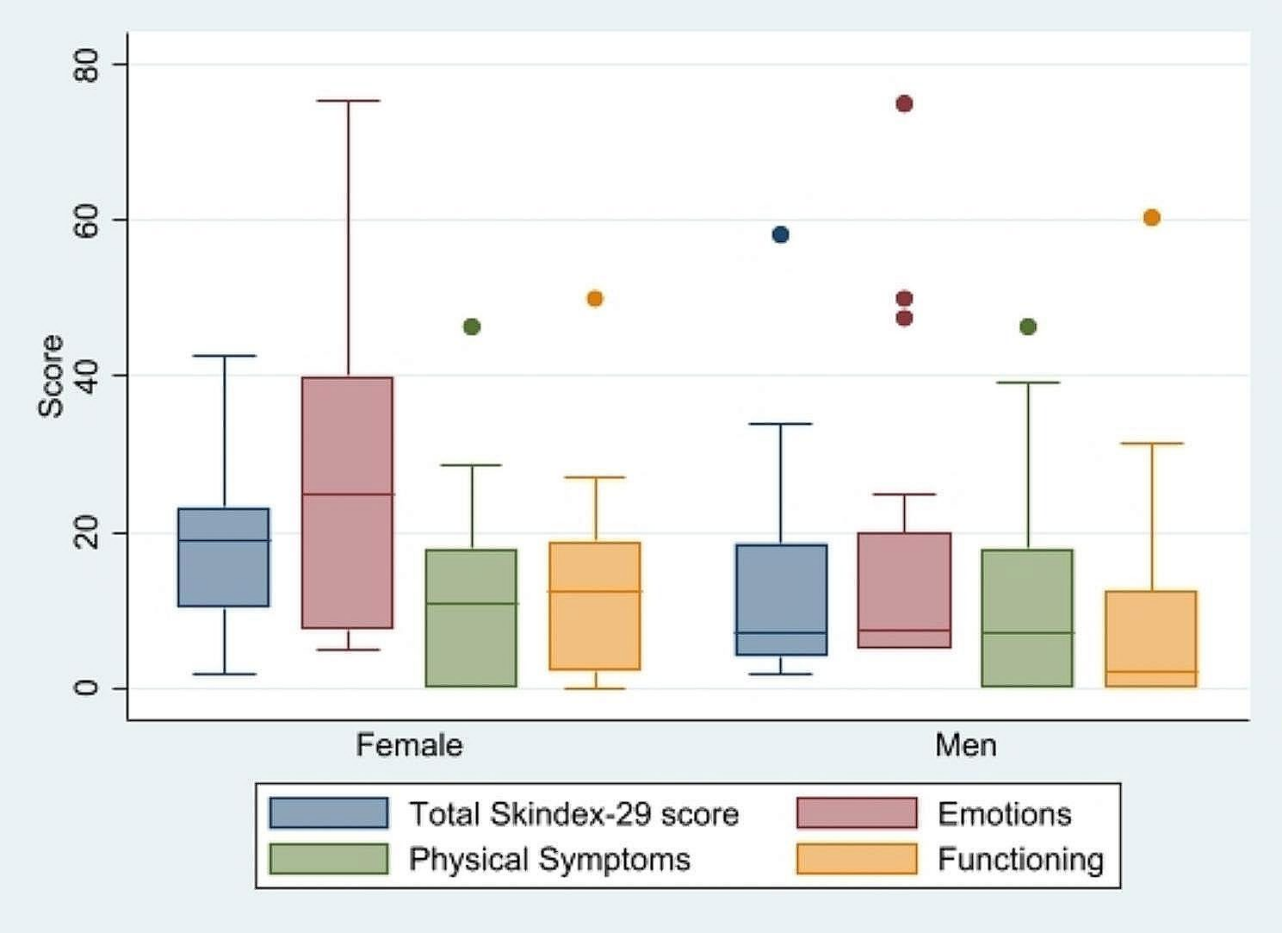
Distribution of mean Skindex-29 scores by sex and domains

Data analysis was performed using SPSS for Windows v.26.0 software (SPSS Inc. Chicago, IL USA). We used non-parametric tests due to the limited number of patients and the non-normal distribution. The data were expressed as median and IQR. For categorical variables, we applied Wilcoxon rank-sum tests and Kruskal-Wallis tests depending on the number of groups to be compared (2 or more than 2). We used Spearman’s rank coefficient correlation for continuous variables. *P* values of < 0.05 were considered as statistically significant. We used STATA16 to create Fig. 2.

## Results

### Sample characteristics

We have clinically evaluated forty patients. The main clinical features and sociodemographic characteristics of the NF1 individuals included in this cohort are summarized in Table 1. Detailed clinical characteristics are available in Table 2.

**Table 1:** Sociodemographic and main clinical features of the NF1 individuals included in this study

		N	n	%
Sex				
	Females	40	21	52.5
	Males	40	19	47.5
Age (years)				
	< 35	40	17	42.5
	35–54	40	14	35.0
	>=55	40	9	22.5
Education level				
	Undergraduate	34	15	44.1
	Vocational education	34	12	35.3
	University degree	34	7	20.6
Clinical characteristics				
	**Six or more café au lait macules**	**40**	**39**	**97.5**
	**Axillary and/or inguinal freckling**	**40**	**38**	**95.0**
	**Two or more neurofibromas**	**40**	**32**	**80.0**
	**Plexiform neurofibroma**	**40**	**17**	**42.5**
	**Two or more Lisch nodules**	**37**	**29**	**78.4**
	**Optic pathway glioma**	**40**	**4**	**10.0**
	**Osseous lesions**	**40**	**20**	**50.0**
	**Family history**	**40**	**20**	**50.0**
	Neurocognitive features	40	18	45.0
	Central Nervous System	40	14	35.0
	Short stature (< P3)	40	14	35.0
	Macrocephaly (> P97)	40	5	12.5
	Emphysema	40	1	2.5
	Pruritus	40	15	37.5
	Hypomelanic macules	40	3	7.5
	Dizziness	40	12	30
	Sleeping troubles	40	10	25.0
	Headaches	40	9	22.5
	Myomas	21	4	19.0
	Malignancies	40	4	10.0

**Table 2:** Detailed clinical information of the 40 NF1 individuals included in this study

ID	Age	Sex	Size	HC	cNF	pNF	CL	F	LN	G	Osseous	FH	Neurocognitive	Education	Others	AS	RS	Genetic variant
1	28	M	165	59.5	0	Sacral plexus	X	X	X	0	Scoliosis	0		Vocational	Hemihypertophia, pruritus	3	3	c.6367G > T, p.E2123X NM_000267.3
2	53	M	169.5	54	X	0	X	X	X	0	Scoliosis	X	TDAH, LD	No studies	Pheocromocitoma, pruritus	2	3	NA
3	34	F	158.5	57	X	Popliteal	X	X	X	0	S. scoliosis	X	LD	Vocational	Ophtalmic aneurysm, myomas, pruritus	3	2	c.4084 C > T; p.(Arg1362*) NM_000267.3
4	60	M	185	62	X	0	X	0	X	0	S. scoliosis	X	0	-	0	2	3	NA
5	22	F	171	60	X	Back	X	X	X	0	0	0	0	University	Dizziness, Wolf-Parkinson-White, pruritus	2	2	c.2379delC,p.(Asn793fs*28) NM_001042492.2
6	21	M	170	60	0	0	X	X	X	0	Scoliosis	X	0	University	Hydrocephaly	1	2	c.7012_7014del, p.(Leu2338del) NM_001042492.2
7	54	M	170	59	X	Para-tracheal	X	NC	0	0	0	0	LD, AD	University	Insomnia	3	3	c.7012_7014del, p.(Leu2338del) NM_001042492.2
8	46	M	174.5	56.5	0	0	0	X	X	0	0	0	TDAH	Vocational	0	1	1	NA
9	80	M	147	58	X	0	X	X	-	0	0	0	0	Primary	Dizziness, headache, pruritus, GIST	2	3	NA
10	22	F	167	52.5	X	0	X	X	0	0	0	0	LD, AD	Vocational	Sleeping troubles, asthenia	1	2	NA
11	21	F	160	57	0	0	X	X	X	0	Scoliosis	0	0	Vocational	Sleeping troubles, asthenia, hypopigmented macules	2	1	NA
12	22	F	160	58	X	Sacral plexus	X	X	0	0	0	0	LD, AD	No studies	Headache	2	3	c.1149 C > A,p.(Cys383*) NM_001042492.2
13	25	M	169.5	58	X	0	X	X	X	0	Scoliosis	X	LD, TDAH	Secondary	0	2	2	NA
14	48	F	154	55	X	0	X	X	X	0	Scoliosis	X	0	Primary	0	3	3	NA
15	34	M	162	59	X	0	X	X	X	0	S.Kypho-scoliosis	X	0	Vocational	Pruritus	2	3	c.574 C > T,p.(Arg192*) NM_000267.3
16	57	M	162	58.5	X	Facial	X	X	X	0	0	0	0	Secondary	Sleeping troubles, headache	3	3	NA
17	17	M	161.5	54.5	0	0	X	X	0	0	0	0	LD, TDAH	Primary	Hypofisis cyst	1	1	Typical 1.4 Mb NF1 deletion
18	51	M	170	59	X	0	X	X	0	0	Kypho-scoliosis	0	LD	Primary	Puritus, Headache, dizziness, meningioma	2	2	NA
19	44	F	163	60	X	0	X	X	X	X	S. scoliosis	0	0	-	Hydrocephaly, pruritus, myomas, sleeping troubles	3	4	NA
20	34	F	155.4	55.6	X	0	X	X	X	0	0	X	AD	-	Hypopigmented macules	1	2	c.6100dup, p.(Thr2034Asnfs*26) NM_001042492.2
21	42	M	191	59	X	Ankle	X	X	X	X	Tibial dysplasia S. scoliosis	0	0	Vocational	Limb length difference, cerebral artery hypoplasia, pruritus sleeping troubles	3	3	c.5606T > G,p.(Leu1869*) NM_001042492.2
22	61	M	165.9	59.3	X	Gluteal	X	X	X	0	S. scoliosis	X	0	-	Dizziness	2	2	NA
23	42	F	172.5	57	X	0	X	0	-	0	0	0	0	University	0	3	2	NA
24	43	M	163	56	X	Right arm	X	X	-	0	S. scoliosis	0	Hyperactivity	Vocational	0	2	2	c.1986delA, p.(Asn664Thrfs*24) NM_001042492.2
25	21	F	151.5	54	X	Sacral plexus	X	X	0	X	Scoliosis	X	0	Vocational	Pruritus, Dizziness	3	4	NA
26	55	F	155	52.5	X	Sacral plexus	X	X	X	0	0	X	LD, AD	No studies	Vertigo	1	2	NA
27	56	F	156	56	X	0	X	X	X	0	0	X	0	-	Pruritus	2	2	c.574 C > T,p.(Arg192*) NM_000267.3
28	43	F	149	56	X	0	X	X	X	0	0	0	0	University	Myomas, pruritus	1	2	NA
29	63	F	144	51	X	0	X	X	X	0	Scoliosis	X	TDAH, LD	No studies	Myomas, sleeping troubles, headache	3	3	c.3826 C > T,p.(Arg1276*) NM_001042492.2
30	53	F	143.5	53	X	0	X	X	X	0	0	X	LD	No studies	Insomnia, asthenia depression, pruritus	3	3	c.3826 C > T,p.(Arg1276*) NM_001042492.2
31	26	F	152.3	50.5	0	Facial, brachial plexus	X	X	0	0	0	X	LD, AD	University	Headache, hypopigmented macules	3	3	NA
32	32	M	173.4	50	X	0	X	X	X	0	0	0	LD, mild ID	Vocational	0	2	3	c.7211del, p.(Ala2404Val*fs*14) NM_001042492.2
33	76	M	154	58	X	Occipital	X	X	X	0	0	0	LD	No studies	Dizziness, pruritus	2	2	NA
34	45	F	148	52.5	X	0	X	X	X	0	S. scoliosis	X	TDAH	No studies	Headache uterine polype, dizziness, depression, pruritus sleeping troubles	3	3	NA
35	35	M	164	61	0	0	X	X	X	0	0	0	0	-	Vertigo	1	1	NA
36	22	M	165.5	57.7	X	Lumbo-sacreal	X	X	0	0	Sphenoid dysplasia	X	0	Vocational	0	3	3	NA
37	58	F	164	55.5	X	0	X	X	X	0	0	X	0	Primary	Ca mama y colon, headache, dizziness, myomas	3	4	c.6792 C > G,p.(Tyr2264*) NM_000267.3
38	28	F	161	58	X	Temporal	X	X	X	X	Sphenoiddysplasia Kypho-scoliosis	X	0	No studies	Dizziness, headache	3	3	c.6792 C > G,p.(Tyr2264*) NM_000267.3
39	29	F	142	54	X	Breast, lumbo-sacreal	X	X	X	0	0	0	0	Vocational	Dizziness, depression, sleeping troubles	2	3	c.6709 C > T,p.(Arg2237*) NM_000267.3
40	35	F	156	56	0	Abdominal	X	X	0	0	C.Scoliosis	X	0	Vocational	0	1	2	NA

### Disease severity/ disease visibility

Results of the Skindex-29 questionnaire, Ablond Index (AI), and Riccardi severity scores (RSS) are summarized in Table 3. Most patients had mild or minimal severity status (52.5%) and moderate disease visibility (42.5%).

**Table 3:** Skindex-29 results in NF1 patients stratified by sex, age group, education level, disease visibility (Ablond’s score) and disease severity (Riccardi’s score)

		N	n	%	Total	Emotions	Physical symptoms	Functioning
Sex								
	Females	40	21	52.5	19.0 (9.3–23.6)	25.0 (7.5–42.5)	10.7 (0.0-19.7)	12.5 (2.1–19.8)
	Males	40	19	47.5	7.1 (4.0-18.6)	7.5 (5.0–20.0)	7.1 (0.0-17.9)	2.1 (0.0-12.5)
	*p value* ^*a*^				0.034	0.061	0.688	0.088
Age (years)								
	< 35	40	17	42.5	10.3 (6.0-21.3)	20.0 (5.0–35.0)	7.1 (0.0-14.3)	4.2 (0.0-18.7)
	35–54	40	14	35.0	19.1 (6.0-25.9)	11.3 (5.0-51.3)	16.1 (0.0-36.6)	6.3 (0.0-20.4)
	>=55	40	9	22.5	12.3 (4.2–21.9)	17.5 (6.3–37.5)	7.1 (0.0-14.3)	10.4 (2.0-15.7)
	*p value* ^*a*^				0.759	0.999	0.209	0.920
Ablond’s score								
	Grade 1	40	12	30.0	9.7 (5.3–20.7)	10.0 (5.0-36.3)	1.8 (0.0-13.4)	2.1 (0.0-15.6)
	Grade 2	40	17	42.5	13.6 (6.6–24.0)	17.5 (6.3–37.5)	7.1 (1.8–23.2)	12.5 (1.1–16.7)
	Grade 3	40	11	27.5	10.3 (2.6–30.6)	10.0 (5.0–50.0)	14.3 (0.0-21.4)	10.4 (2.0–25.0)
	*p value* ^*b*^				0.545	0.825	0.207	0.397
Riccardi’s score								
	Grade 1	40	5	12.5	6.4 (5.8–9.7)	5.0 (5.0–15.0)	10.7 (3.6–14.3)	2.1 (0.0-9.4)
	Grade 2	40	16	40.0	11.0 (4.3–22.4)	15.0 (5.0–25.0)	3.6 (0.0-23.2)	3.2 (0.0-14.1)
	Grade 3	40	15	37.5	18.6 (8.8–33.8)	25.0 (7.5–50.0)	14.3 (0.0-21.4)	12.5 (2.1–25.0)
	Grade 4	40	4	10.0	13.4 (3.3–27.7)	21.3 (5.0-48.8)	10.7 (1.8–14.3)	8.4 (1.1.21.9)
	*p value* ^*b*^				0.202	0.173	0.876	0.130

^a^ Contrast data by Spearman rank correlation. ^b^ Contrast data by Kruskal-Wallis.

### Skindex-29

The median of Skindex-29 scores was 12.3 (IQR 5.9–22.4). Emotions items (median 15.0; IQR 5.0-37.5) scored higher than symptoms (median 8.9; IQR 0.0-17.9) and functioning (median 8.3; IQR 0.5–18.3). Figure 2 shows the results stratified by sex and domain (emotions, physical symptoms, and functioning). We observed an association between the emotion and functioning scores (*p* < 0.001) and between the symptoms and the functioning scores (*p* = 0.042). The 5 items with a higher score were Q13 “I worry that my skin condition might get worse” (85 points, emotion), Q12 “I am ashamed of my skin condition” (47 points, emotions), Q6 “My skin made me feel depressed” (44 points, emotion), Q5 “My skin affects my social life” (42 points, functioning), and Q10 “My skin itches” (40 points, symptoms).

Women showed higher scores on the Skindex-29 questionnaire (women: median 19.0; IQR 9.0-23.6 and men: median 7.1; IQR 4.0-18.6; *p* = 0.034) than men. We observed a trend for sex differences in emotion (woman: median 25.0; IQR 7.5–42.5 and men: median 7.5; IQR 5.0–20.0; *p* = 0.061) and functioning items women: median 12.5.; IQR 2.1 − 19.8 and men: median 2.1; IQR 0.00–12.50; *p* = 0.088). We did not observe sex differences for symptoms, disease severity, or disease visibility scores. Aging did not correlate with increased scores on emotions, functioning, or physical symptoms.

Fifteen NF1 individuals (37.5%) complained of itching. The presence of pruritus was associated with higher scores on the symptoms domain of the Skindex-29 questionnaire (p˂0.001). The sleep troubles described in 10 NF1 individuals (25%) significantly impacted the Skindex-29 scores on the functioning (*p* = 0.013) and symptoms domains (*p* = 0.047).

## Discussion

Improving the QoL of our patients is an essential objective for all physicians and health workers. This objective is even more important in patients with rare disorders as they must frequently deal with ignorance and incomprehension.

Several QoL questionnaires have been used and developed worldwide for the NF1 population. However, standardization is needed to compare the QoL of NF1 patients from different regions or countries. There are only a few studies that have assessed QoL in NF1 patients with the Skindex-29 questionnaire: in France [27], Italy [10], the USA [18], and Australia [7, 28, 29]. Compared to prior studies, we observed a lower impact of NF1 in the QoL of the patients described in this study, as their scores were lower than in earlier reports. The emotional dimension was significantly more impacted than the other dimensions, consistent with previous studies. The different impacts of NF1 in those studies could reflect differences in enrollment, population, disease staging, demographic differences, health care system, and study design. All NF1 individuals included in this study were identified from a primary care unit or reference hospital in a semi-urban area where patients are followed mainly by general practitioners and dermatologists and may, therefore, be more representative of the general population than patients in a Neurofibromatosis clinic. Furthermore, Spain has a public health system, which might contribute to facilitating access to medical care and lessen the financial burden of the disease and the impact on disease perception. We performed the clinical exam and administered the Skindex-29 at the same time point. Thus, Ablon’s visibility and Riccardi’s severity scores were assessed by physicians and not by the patient himself who could be biased by his own disease perception. However, the main limitation of this study is the small number of NF1 patients enrolled, which limited the statistical power of some findings.

The Skindex-29 questionnaire revealed that women with NF1 are more severely impacted than men. Sex differences in QoL in NF1 patients were previously observed [8, 18, 21]. Women with NF1 have higher scores on emotion, perceived physical appearance, anxiety, and mental health [8]. We also observed a strong association between emotions and functioning scores on Skindex-29, which might indicate that both domains are highly related. Therefore, clinicians should be more attentive to the emotional status of NF1 patients and be more prone to use depression-screening questionnaires such as the PHQ-9 [30, 31] or the Generalized anxiety disorder-7 item scale (GAD 7) [31, 32]. Depression has been described in up to 55% of NF1 [33] patients and is associated with pain intensity and pain interference [34, 35]. A high occurrence of suicide ideation was previously described in NF1 individuals attributable to several psychosocial factors associated with NF1, including depression, anxiety, perceived stress [9, 36], pain, and QoL domains [37, 38]. Improvement of the QoL and emotional status of NF1 individuals is a critical unmet need. Several articles have shown a clear benefit for NF1 individuals to perform face-to-face [39] or online [40] mind-body-based interventions which can be associated or not with pharmacological treatments [41] to improve their emotional status and QoL. Recently, a Relaxation Response Resiliency Program (3RP-NF) including mindfulness, copying, and optimism interventions has shown a durable improvement in QoL among adults with NF1 [42, 43]. Therefore, psychosocial interventions in NF1 individuals should be recommended in the NF1 follow-up protocols to be able to apply for cost coverage/reimbursement by the different health systems worldwide. Otherwise, NF1 individuals with lower income status and at higher risk of psychological burden would be disadvantaged.

As expected, the frequency of the main clinical features of NF1 patients enrolled in this study was like previous literature reports [1, 44, 45]. However, it is interesting that about 1/3 of the patients complained of pruritus and ¼ dizziness, headache, or sleeping troubles. Although those are not life-threatening, they have impacted NF1 patient’s QoL. Pruritus has been described in about 35–69% of patients in prior studies [29, 46] and was described as the more bothersome symptom of NF1 in 14% of patients [2]. Pruritus seems to be associated with the development of new neurofibromas [29]. However, the pathophysiology of pruritus is not well-understood and seems to be complex. Pruritus seems to have neuropathic features [47] but could also be explained by the degranulation of the mast cells which are merged into the cutaneous neurofibromas [48]. There are currently no established guidelines for the treatment of itching in NF1. Doctors treat most patients with emollients, steroid creams, or antihistamines [29] but gabapentine (an antineuropathic agent) or ketotifene (a mast cell stabilizing agent) has also been used. When itching is localized in one or 2 neurofibromas, neurofibroma removal by surgery, carbon dioxide laser [49, 50], or electrodesiccation [51] is considered.

Migraines and non-migraines headaches are frequent in NF1 patients [52, 53]. Migraine can be observed in 34 to 83% of patients with NF1, significantly affecting their QoL [52, 53]. Therefore, patients should receive specific anti-migraine treatment. Sleep troubles such as parasomnias, difficulties in initiation sleep, early morning awakenings, and excessive sleep/wake transition are also frequently described in NF1 patients [54]. A sleep study on 114 NF1 patients identified 69% as being “poor sleepers” and 20% with excessive day sleepiness [54]. As sleep disturbance is a widespread migraine trigger, it might be responsible for some of the headaches described in our patients and may predispose them to depression. Therefore, NF1 patients should benefit from the classical diagnostic and therapeutic strategies [55].

## Conclusion

This study highlights the impact of NF1 in the QoL in a cohort of patients from a semi-rural public health primary care area. NF1 shows an essential effect on the emotional status of NF1 patients, which would justify implementing self-esteem strategies to prevent mental health in at-risk individuals. Thus, we identified itching, and sleep troubles that negatively influence the QoL of NF1 patients. Since patients do not perceive these symptoms as NF1-related, patients may not mention them. Therefore, clinicians should actively seek those symptoms to treat them to improve the QoL of their NF1 patients.

### Electronic supplementary material

Below is the link to the electronic supplementary material.

Supplementary Material 1

## Data Availability

All data supporting the findings of this study are available within the paper and its Supplementary Information.

## Abbreviations

QoL: Quality of life
NF1: Neurofibromatosis type 1
